# Diffuse Alveolar Hemorrhage as the Initial Manifestation of Acute Myelomonocytic Leukemia

**DOI:** 10.1155/crh/3835062

**Published:** 2026-04-22

**Authors:** Samyukta Varma, Abdullah Al Twal

**Affiliations:** ^1^ Department of Internal Medicine, Marshfield Medical Center, Marshfield, Wisconsin, USA; ^2^ Department of Pulmonology and Critical Care, Marshfield Medical Center, Marshfield, Wisconsin, USA

## Abstract

Diffuse alveolar hemorrhage (DAH) is a rare but life‐threatening condition caused by the accumulation of red blood cells in the alveolar spaces, leading to respiratory failure. While DAH has been reported in patients with acute myeloid leukemia (AML) following chemotherapy, its occurrence as an initial manifestation of AML is uncommon. We present the case of a 31‐year‐old female with no known hematologic history who presented with hemoptysis and dyspnea. Her leukocyte count was 28.2 × 10^9^/L with 83% monocytic predominance. The patient underwent diagnostic bronchoscopy, which confirmed DAH. Bone marrow biopsy revealed AML with monocytic differentiation. Flow cytometry showed a positive inversion 16, and FLT3 mutation was negative. The patient was treated with induction chemotherapy (7 + 3 regimen: daunorubicin and cytarabine), but her condition deteriorated, and she eventually succumbed to respiratory failure. This case highlights the importance of considering hematologic malignancy in the differential diagnosis of DAH, particularly in the absence of other identifiable causes.

## 1. Introduction

Diffuse alveolar hemorrhage (DAH) is a life‐threatening condition marked by the accumulation​ of red blood cells within the alveolar spaces due to disruption of the alveolar‐capillary membrane, resulting in respiratory failure. Clinically, DAH manifests with symptoms such as dyspnea, hemoptysis (absent in approximately one‐third of cases), diffuse pulmonary infiltrates on imaging, and a swift decrease in hemoglobin levels [1–3].

The causes of DAH are varied, including primary and secondary vasculitis, systemic autoimmune disorders (e.g., systemic lupus erythematosus), hematologic malignancies, and complications following hematopoietic cell transplantation [1, 4–6]. In vasculitis, DAH is commonly caused by capillaritis [1]. In the context of hematopoietic cell transplantation, DAH is associated with conditioning regimens, graft‐versus‐host disease, and infections [4].

Diagnosis is primarily based on clinical presentation, imaging, and bronchoalveolar lavage (BAL), which typically reveals progressively hemorrhagic aliquots [1, 4]. Lung biopsy, while conclusive, is generally avoided due to its invasive nature.

Treatment strategies for DAH include supportive care, high‐dose corticosteroids, and immunosuppressive agents such as cyclophosphamide and rituximab, particularly in cases associated with vasculitis [1, 5]. In severe cases, mechanical ventilation and extracorporeal membrane oxygenation (ECMO) may be necessary [1]. For life‐threatening vasculitis‐associated DAH, such as antiglomerular membrane disease or severe systemic lupus erythematosus (SLE), therapeutic plasma exchange and intravenous immune globulin (IVIG) may be considered, although data supporting these interventions remain limited [1].

DAH is a potentially fatal complication in patients with AML. While DAH more commonly occurs after the initiation of chemotherapy, its occurrence as an initial manifestation of AML is rare but clinically significant. Here, we present a case of acute myelomonocytic leukemia (AMML) initially presenting as DAH.

CARE reporting statement: This case report has been prepared in accordance with the CARE (CAse REport) reporting guidelines. The completed CARE checklist is provided as Supporting Information (available here).

## 2. Case Presentation

**FIGURE 1:**
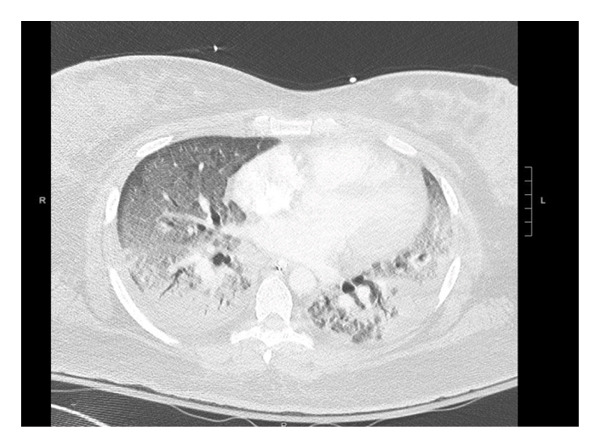
CTA chest showing diffuse, patchy infiltrates throughout both lungs.

**FIGURE 2:**
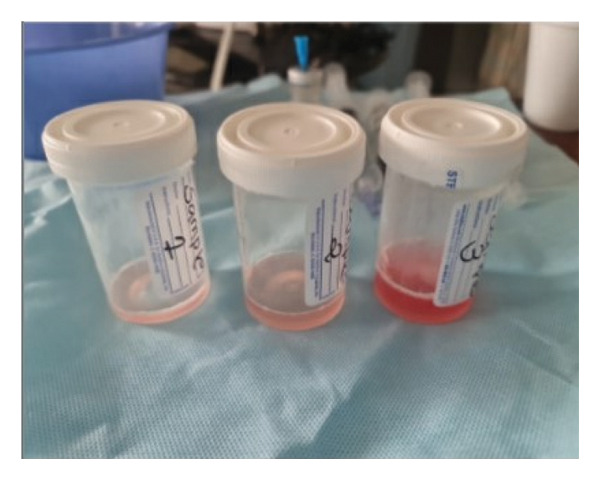
Progressively hemorrhagic serial lavage aliquots.

A 31‐year‐old female with a medical history of ADHD, GERD, prediabetes, mood disorder, and polycystic ovarian disease (PCOD) presented with a 1‐month history of fatigue and productive cough with greenish sputum. Her symptoms worsened, culminating in hemoptysis and dyspnea, prompting ER evaluation. On presentation, her vitals were remarkable for a heart rate of 122/minute, a blood pressure of 148/80; she was afebrile, with a respiratory rate of 20 per minutes, saturating at 98% on room air. Initial labs showed a white blood cell count of 28.2 × 10^9^/L, with 83% monocytic predominance, a hemoglobin level of 8.9 g/dL, and a platelet count of 82 × 10^3^/L. Chest CT angiography (CTA) ruled out pulmonary embolism and revealed diffuse bilateral pulmonary infiltrates (Figure 1). Patient was initially admitted to the general medical ward; however, she had worsening hemoptysis with increasing oxygen requirements and subsequently was transferred to the medical intensive care unit (ICU) where she underwent diagnostic bronchoscopy, which confirmed the diagnosis of DAH, with BAL taken from the right middle lobe revealing progressively hemorrhagic serial lavage aliquots (Figure 2).

An extensive infectious, vasculitis, autoimmune, and coagulopathy workup was unremarkable. BAL studies did not demonstrate any evidence of bacterial, viral, or fungal infections. An echocardiogram was performed that showed a normal right ventricular systolic function and an estimated normal right ventricular systolic pressure of 31 mm Hg. Given the clinical context and marked monocytic predominance in both peripheral blood and BAL (BAL differential with monocytes 61%, neutrophils 35%, lymphocytes 1%, eosinophils 1%, and basophils 1%), leukemic infiltration of the lungs was considered the most likely cause of DAH.

A bone marrow biopsy revealed AML with monocytic differentiation. Flow cytometry showed a positive inversion 16, and FLT3 mutation was negative. The patient was started on induction chemotherapy with the 7 + 3 regimen consisting of daunorubicin and cytarabine. Prophylactic measures for tumor lysis syndrome and opportunistic infections were initiated.

Despite aggressive supportive care, the patient’s respiratory status continued to deteriorate, and she progressed to acute respiratory distress syndrome (ARDS), requiring endotracheal intubation and mechanical ventilation. Her ICU course was prolonged and complicated, and by hospital day 20, she experienced further decompensation despite maximal ventilatory support. After goals‐of‐care discussions with the family, the decision was made to transition to comfort care. The patient was compassionately extubated and subsequently passed away. Postmortem examination was not performed; therefore, no autopsy data were available to further characterize the pulmonary pathology.

## 3. Conclusion

In the context of AMML, DAH can be precipitated by several contributing factors, including thrombocytopenia, coagulopathy, and the immunocompromised state associated with both the malignancy and its treatment [7]. Patients with AMML, particularly those with KMT2A rearrangements, are at higher risk for developing DAH. One study reported that 42% of patients with KMT2Ar AML who died within 60 days had a diagnosis or strong clinical suspicion of DAH, compared to 18% of those with normal karyotype AML [8]. Although KMT2A rearrangement status was not assessed in our patient, inv(16) AML and KMT2A‐rearranged AML are considered distinct molecular subtypes and are generally mutually exclusive, making a concurrent KMT2A rearrangement unlikely in this context.

Given that AML can present with various pulmonary complications—including leukostasis, leukemic infiltration of the lung, and acute lysis pneumopathy—a high index of suspicion for AML should be maintained in patients presenting with DAH after infectious or vasculitic etiologies have been ruled out [9].

The American Society of Clinical Oncology (ASCO) and the College of American Pathologists recommend a thorough diagnostic workup in suspected acute leukemia cases, including peripheral blood smear, bone marrow aspirate, and cytogenetic analysis, to confirm the diagnosis and guide appropriate treatment decisions [10].

In summary, although DAH is an uncommon initial manifestation of AML, it is crucial to consider AML in the differential diagnosis of DAH—particularly in the absence of other identifiable etiologies. Prompt recognition and comprehensive diagnostic evaluation are critical for timely intervention and improved patient outcomes.

## Author Contributions

Samyukta Varma contributed to patient care, data collection, manuscript drafting, and revision. Abdullah Al Twal contributed to clinical management, critical revision of the manuscript, and supervision.

## Funding

No funding was received for this study.

## Disclosure

All authors have read and approved the final version of the manuscript. Samyukta Varma had full access to all the data in this study and takes complete responsibility for the integrity of the data and the accuracy of the data analysis.

## Ethics Statement

No IRB approval was required for this single case report.

## Conflicts of Interest

The authors declare no conflicts of interest.

## Supporting Information

CARE reporting guidelines: This case report has been prepared in accordance with the CARE reporting guidelines. The completed CARE checklist has been provided as a supporting file.

## Supporting information


**Supporting Information** Additional supporting information can be found online in the Supporting Information section.

## Data Availability

Data sharing is not applicable to this article as no datasets were generated or analyzed beyond the clinical information presented in this case report.
