# Supplementary material for: Diffuse Alveolar Hemorrhage as the Initial Manifestation of Acute Myelomonocytic Leukemia
Source: Case Rep Hematol. 2026 Apr 22;2026:3835062. doi: 10.1155/crh/3835062 (PMC13100640; doi:10.1155/crh/3835062)
Supplement: Supplementary file 1 — Supporting Information Additional supporting information can be found online in the Supporting Information section. [file CRH-2026-3835062-s001.docx]

CARE Checklist – Case Reports in Hematology

Manuscript Title: Diffuse alveolar hemorrhage as the initial manifestation of Acute Myelomonocytic Leukemia

- Article Type: Single Case Report
- Title – Identifies the article as a case report
- Abstract – Structured summary including background, case presentation, and conclusion
- Introduction – Brief background and rationale
- Patient Information – Demographics and clinical history
- Clinical Findings – Relevant examination findings
- Timeline – Chronological summary of events
- Diagnostic Assessment – Laboratory, imaging, and cytogenetic findings
- Therapeutic Intervention – Treatment details
- Follow-up and Outcomes – Clinical course and outcome
- Discussion – Strengths, limitations, and literature comparison
- Patient Perspective – (Not applicable)
- Informed Consent – Obtained / IRB not required for single case report

This checklist confirms adherence to the CARE reporting guidelines.
